# Self-care habits among people who inject drugs with skin and soft tissue infections: a qualitative analysis

**DOI:** 10.1186/s12954-019-0345-z

**Published:** 2019-12-12

**Authors:** Andrew R. Gilbert, Julia L. Hellman, Michael S. Wilkes, Vaughan W. Rees, Phillip J. Summers

**Affiliations:** 1 Transitions Clinic, Sacramento, CA 95817 USA; 2 Harvard T. H. Chan School of Public Health, Boston, MA 02115 USA; 3 UC Davis School of Medicine, Sacramento, CA 95817 USA; 4 Center for Global Tobacco Control, Department of Social & Behavioral Sciences, Harvard T.H. Chan School of Public Health, Boston, MA 02115 USA

**Keywords:** Injection drug use, Heroin, Skin and soft tissue infection, Self-care, Barriers to healthcare, Access to healthcare, Harm reduction, Qualitative methods

## Abstract

**Background:**

Injection drug use is on the rise in the USA, and skin and soft tissue infections (SSTI) are a common complication, resulting in significant morbidity and mortality. Due to structural barriers to care-seeking, many people who inject drugs avoid formal care and resort to self-care techniques, but little is known about the nature of these techniques, or more generally about the accuracy or breadth of this population’s knowledge of SSTIs.

**Methods:**

Semi-structured qualitative interviews were conducted with 12 people who inject heroin in two metropolitan areas: Sacramento and Boston, USA.

**Results:**

These interviews reveal a robust and accurate knowledge base regarding skin infections, including the progression from simple cellulitis to an abscess, and acknowledgment of the possibility of serious infections. Nonetheless, there remains a reticence to seek care secondary to past traumatic experiences. A step-wise approach to self-care of SSTI infections was identified, which included themes of whole-body health, topical applications, use of non-prescribed antibiotics, and incision and drainage by non-medical providers.

**Conclusions:**

The reported SSTI self-care strategies demonstrate resilience and ingenuity, but also raise serious concerns about inappropriate antibiotic consumption and complications of invasive surgical procedures performed without proper training, technique, or materials. Harm reduction agencies and health care providers should work to obviate the need for these potentially dangerous practices by improving healthcare access for this population. In the absence of robust solutions to meet the needs of this population, education materials should be developed to optimize the efficacy and minimize the harms of these practices, while empowering and supporting the autonomy of people who use drugs and providing clear guidance on when self-care should be abandoned in favor of formal medical care.

## Introduction

The U.S. Department of Health and Human Services has declared a public health emergency [12] regarding the opioid crisis, with 46 out of 50 states seeing an increase in opioid-related deaths from 2010 to 2016 [7]. The two most commonly used forms of opioids are heroin and prescription pills. Injection drug use (IDU) is a common route of administration for heroin, and in 2016, approximately 948,000 Americans reported using heroin in the past year, while approximately 2.2 million people in the United States reported injecting heroin at least once in their life [19]. For unclear reasons, heroin confers a higher risk of bacterial infections compared to those who inject amphetamines and those who inject multiple substances [6]. Among this population, skin and soft tissue infections (SSTIs), including cellulitis and abscesses, are the most common reasons for hospitalizations [2, 8, 22], and rates of opioid-related SSTIs are increasing in the US, up to 9 per 100,000 in 2010 [3, 5, 23]. Untreated SSTIs pose significant risks, including endocarditis, septicemia, and necrotizing fasciitis, which increases morbidity and healthcare costs for this population [3, 10].

Despite these significant risks, people who inject drugs (PWID) avoid seeking treatment for all types of care at greater rates than people who do not inject drugs [4, 11]. Commonly reported reasons for delaying treatment include stigma and discrimination from health care providers [1]. Our previous research has shown that PWIH often delay care and resort to treating SSTIs outside of the formal healthcare system, and that fear of inadequate pain control and withdrawal played a significant role in their decisions to delay or avoid seeking care [9, 26]. In this study population, 38% report delaying care for SSTI by 2 weeks or more, 57% left the hospital against medical advice, 54% had lanced their own abscesses, and 32% reported taking non-prescribed antibiotics [26]. The reticence to pursue formal health care forces PWIH to turn to themselves and trusted community members for care. Historically, PWIH have incised their own abscesses, sought out non-prescribed antibiotics, and participated in various forms of homeopathic care [9, 20]. This is particularly salient in the context of a population of PWIH who experience a high burden of disease with poor access to formal healthcare channels [6]. Despite the ubiquity of these phenomena, PWID’s perspectives and understanding of SSTI pathophysiology, risks, treatment options, and self-care behaviors have not been qualitatively described in the literature to date. The concept of self-care for SSTI among PWIH became an unanticipated prominent theme during our initial qualitative analysis of these data, and ultimately inspired this secondary analysis.

Using an approach that is both theory-guided and informed by our recent empirical findings, we therefore sought to understand how PWIDs understand and care for their SSTIs, using a secondary analysis of qualitative data collected as part of this larger mixed-methods study, which was previously published [25, 26]. The theoretical model we employed is based on two previously described models describing health-seeking behaviors. The health belief model (HBM) of health-seeking behaviors describes perceived severity, susceptibility, benefits, barriers, and cues to action [15]. Perceived barriers are the most powerful dimension of this model, though no studies to date explore HBM in the context of PWID. The Conceptual Model of Medical Care Avoidance proposed by Taber et al. [28] based on National Cancer Institute data and the Crisis Decision Theory [27] describes the response to negative events (i.e., a health event) as “first by appraising the severity of threat, next by identifying available response options, and lastly by evaluating available response options.” This creates a construct where health care need is defined based on one’s conceptualization of their health status and the nature of the condition at hand (i.e., SSTI), while recognizing that certain barriers exist, so alternatives are sought in the context of aversion to and/or inaccessibility of formal medical care. These themes are qualitatively explored to elucidate PWID’s understanding of SSTIs and approaches to addressing them.

## Methods

### Setting and design

This qualitative analysis was developed from a parent mixed-methods study using the Priority-Sequence Model. We performed semi-structured interviews conducted with 12 PWIHs (included in this analysis) and a structured survey administered to 145 PWIHs (not included in this analysis), as reported previously [25, 26]. Participants were recruited from two urban areas in the USA: greater Boston, MA, and Sacramento, CA, USA. Participants were split equally between the two sites with 6 interviews conducted in each city. Once the themes were identified, following these key informant interviews, the survey questions were expanded in response to this inquiry in order to understand trends and provide confirmation of the qualitative findings in the greater population of PWIH in these cities. This study’s research question emerged from this qualitative and quantitative data, and the targeted secondary qualitative analysis was then performed to address these new research questions regarding self-care.

### Qualitative methods

The researchers gained access to the study population after collaborating with harm reduction agencies in two locations, building mutual trust and understanding with staff and clients. The study was conceptualized, designed, and implemented with feedback and guidance from the staff and participants at these partner agencies. In particular, the harm reduction staff were instrumental in identifying a purposive sample and facilitating rapport with potential participants. The two cities, Boston and Sacramento, were chosen due to high rates of injection drug use and unique demographics and drug supplies present in each location. As previously reported, compared to PWIH in Boston, where “powder” heroin is most common, PWIH in Sacramento predominantly use “black tar” heroin and have substantially higher rates of vein loss and SSTI [25]. The 12 interviews were conducted with a purposively selected sample by two researchers using a semi-structured interview script. The interviews covered injection practices, personal experiences and understandings of skin infections, and behaviors around care of skin abscesses, and experiences interfacing with healthcare systems. Our sampling strategy maximized information-rich cases within the confines of our study resources by purposefully seeking a sample of diverse experiences and perspectives. Participants were included if they were actively using heroin based on self-report. This allowed us to explore topics of interest that were unique to PWID who are dependent on opioids: specifically, withdrawal and difficulty with acute pain control due to opioid tolerance. Self-care strategies for SSTI emerged spontaneously during nearly all of the interviews, and particularly those in Sacramento where SSTI rates were higher. Due to the mixed methods design of this research and the limited capacity of the researchers, the number of participants was limited to 6 at each site for a total of 12. As this was a retrospective secondary analysis, the cohort was not expanded to reach saturation on this theme. Participants were excluded if they did not speak English or reported that they were under 18 years of age. Prior to obtaining verbal consent, participants were informed on the purpose and components of the study using a standardized consent script approved by the IRB, and were assured that all responses would remain confidential.

Interviews lasted approximately 1 h, were conducted in a private room within the harm reduction agencies, recorded digitally, and transcribed verbatim by the researchers. Transcriptions were sent to a third researcher for coding and category formation using a general inductive approach, in which analysis is determined both by the research objectives (deductive) and through familiarity and analysis of the raw data itself (inductive). This approach is useful for condensing varied, raw data into categories and themes, which can then be linked to research objectives and, ultimately used to develop theories about the underlying processes [29]. Transcripts were read several times to identify themes and categories, after which a coding frame was developed, and the transcripts were coded according to this frame. Coding was performed using color codes and memoing in Microsoft Word and Excel. As new codes emerged, the coding frame evolved and transcripts were reread according to this structure. Coding stopped once no codes emerged. At this point, the iterative process was used to develop broader categories, which, through discussion, were expanded into larger, key themes. Through this iterative process of developing codes, categories, and themes, a Thematic Analysis approach was employed to develop the findings presented below [30]. As the interviews were performed during a single, limited time period, saturation was not specifically achieved through ongoing interviews.

### Informed consent

For all participants, verbal informed consent was obtained using a standardized script. No identifying or contact information was collected from any participant. Any questions or concerns expressed by the participants were addressed at the end of the session, and needle exchange staff were consistently available for any additional education or services requested. Participants were provided with a $5 gift card to a local chain pharmacy. This study protocol was approved by the IRBs of both the Harvard T. H Chan School of Public Health and the University of California, Davis Health System.

## Results

Participant characteristics of the interview participants are summarized in Table 1. The mean age was 46, and there is a slight predominance of white males.

**Table 1:** Interview participant characteristics

Location	Age (years)	Sex	Ethnicity/Race
Boston	37	Male	White
Boston	58	Female	Black and American Indian
Boston	36	Male	White
Boston	62	Male	White
Boston	61	Male	White
Boston	37	Male	White
Sacramento	42	Male	White
Sacramento	37	Female	White
Sacramento	56	Male	Black
Sacramento	49	Male	Hispanic
Sacramento	42	Female	White
Sacramento	36	Male	Hispanic
Ratio 1:1	Mean age = 46	75% Male	67% White

Participants accurately described abscesses, and their various stages of progression, including serious illness and possible death. Notably, they determined that there is a critical point at which that abscess needed to be drained and supportive measures would not be helpful. Participants identified many risk factors for SSTI, including accidental subcutaneous injection and poor personal and injection hygiene practices. Participants described multiple self-care techniques practiced at various stages of an evolving infection, and most were consistent between participants. Common self-care practices include obtaining antibiotics from various non-traditional sources, improvised wound care, needle aspiration, and incision and drainage. Multiple factors played into participants’ decision to pursue informal versus formal care, prominently including positive and negative perceptions of health care experiences, frequency of past abscesses, and firsthand or secondhand experiences with life-threatening complications of skin and soft tissue infections. Themes emerging from participant responses are organized and summarized in Table 2.

**Table 2:** Summary of qualitative findings

Category	Theme	Example quote
SSTI description	Progression of SSTI from cellulitis to abscess	An abscess is an infection that is under your skin, that is full of pus, and need to be cut open and drained.
	Progression to “point of no return”	I wait until they get really big and the surface skin is bulging out and its all red, except for the tip is white where it’s the thinnest.
SSTI causes	Missed injections	I missed a portion of the shot into my arm. I missed the vein, so that was the reason for the infection.
	Hygiene and sanitation	Not using clean supplies. I think my problem maybe if I have not just showered, if I’ve been working all day.
		I’ve seen people not use alcohol wipes. You cannot do that. You cannot, you know, get ready to shoot yourself in the arm and not use an alcohol wipe.
Signs of a SSTI	Presentation of early SSTI symptoms	It gets red and it gets hot and it hurts. It gets to a point when you just cannot bear it any more.
		Because I’ve seen it when it’s blown up or whatever and they cannot even more their hand and stuff like that anymore. That’s when it’s become so infected.
Early SSTI treatment	General health and well-being practices	Little things like drink a lot of liquids, make sure you sleep every night. Make sure you get enough sleep, drink liquids, eat regularly. These are key points.
	Application of hot compress and salve	It all depends. If you catch it right off the bat, you can get some type of a hot pad or something, and put it on there. You continue to do that procedure.
		My thing, I swear by it, draw-out salve.
Late SSTI treatment	Antibiotics from nontraditional sources	You can go into the aquarium, the fish store. They have antibiotics there that are for the fish. And they will go and they will get them and they will take them.
		I sold antibiotics.
	Wound care	I use wet-to-dry bandages with another bandage on the top of it. It’ll help it drain it and it will debride the dead tissue when you pull it out three times a day.
	Needle aspiration	A lot of people will take needles and put them in there and try to drain the fluid out like that.
	Incision and drainage procedure	I would lance it and squeeze it. I pull the skin apart as I’m pushing down on it.
		I think it’s called expression? To open it up, to try and get all the material out. I cannot remember the term … Irrigate. People use a large syringe, preferably with the cleanest water they can get.
Traumatic past experiences within the formal health care system	Psychological trauma	He was mean and cold and short with me. I felt like he did not want to touch me.
		I did not get treated very well at all. It was almost like that attitude that ‘You put yourself here. What are you going to expect us to do? I hope you do suffer’
	Physical trauma	Incorrect treatment, when they cut a larger part than they needed to, mainly to increase pain...
		...and literally made it worse than it had to be. You know, inflicted as much pain as they could. By not administering anesthetic and not enough of what they did, and then just grinding away at you in a way that was very unprofessional. ‘Maybe this will teach you a lesson about using drugs’.

### Description of an abscess

Many participants were able to describe a SSTI accurately in practical and common terms. They routinely described an SSTI as an infection forming under the skin. An important component of this understanding was the knowledge that once the abscess cavity is formed, it is toxic or “poisonous,” antibiotics are insufficient, and the wound must be surgically drained or the person risks complications like septicemia and endocarditis

An abscess is an infection that is under your skin, that is full of pus, and needs to be cut open and drained.

- 37-year-old woman

An abscess is an infection of the surface skin, um, around an injection site.

- 37-year-old man

...it could be a build up of liquid...But it becomes poison inside of you. If you go without getting it taken care of, it can become more and more and more serious...The poison starts traveling through your body, and it can travel to your heart....so they’ll give you some antibiotics and treat it. Sometimes...they cut into your body where the abscess is at, and drain all that poison out.

- 56-year-old man

### Causes of an abscess

Participants expressed two common themes regarding the etiology of SSTIs: “missed” injections and unhygienic practices.

Most participants expressed intravenous injection as the preferred route of administration. The theme of “missing” intravenous injections—resulting in inadvertent subcutaneous injection—was correctly identified as a potential risk of SSTI formation [14], and lamented as an unfortunately common event.

I missed the vein, so that was the reason for the infection. I think that’s the reason for a lot of infections. I don’t get infections when I don’t miss the vein.

- 42-year-old man

Most likely you missed though, I’ve never really seen anyone get an abscess that hits because you miss and just like the stuff accumulated under the skin.

- 37-year-old man

All I know is that I put it in and blood came out and I pushed it back in and I must have missed putting it back in and it goes underneath the skin and it makes a lump.

- 58-year-old woman

A second common theme identified among interview participants was poor hygiene and sanitation practices while injecting was another common theme identified by interview participants.

The last abscess I had, it was an issue of lack of sanitation.

- 42-year-old man

Not using clean supplies. I think my problem maybe if I haven’t just showered, if I’ve been working all day.

- 37-year-old woman

I’ve seen people not use alcohol wipes. You can’t do that. You can’t, you know, get ready to shoot yourself in the arm and not use an alcohol wipe.

- 42-year-old woman

The main issue would be improper sanitation and not washing their hands, and not using their materials like a spoon, etc, not using clean ones. That’s a major issue right there.

- 42-year-old man

### Signs of an abscess

The clinical presentation of a SSTI includes erythema, warmth, edema, and pain over the affected site, systemic features of infection may follow as well [24]**.** Participants were able to correctly list these symptoms. Many also described the chronology of symptoms, noting a vaguely-defined “point of no return,” prior to which an abscess will resolve spontaneously, and after which an abscess requires treatment to prevent life- or limb-threatening complications.

It gets red and it gets hot and it hurts. It gets to a point when you just can’t bear it any more. You just can’t walk. Or you can’t use wherever it is on your body, you can’t use that limb. You can’t use your arm. You can’t use your leg or your foot. It becomes to where it’s like affecting you and you can’t function.

- 37-year-old woman

Because I’ve seen it when it’s blown up or whatever and they can’t even more their hand and stuff like that anymore. That’s when it’s become so infected.

- 36-year-old man

Many participants also described the progression from cellulitis (which cannot be drained) to an abscess, which requires drainage in some capacity.

I wait until they get really big and the surface skin is bulging out and it’s all red, except for the tip is white where it’s the thinnest. And I feel around on it to feel where it’s still soft and where it’s still … around the edges your tissue is swelled up. It will swell up like this [makes doughnut shape with hand] with a little lump and a ring around it. I want to check on the inside of the ring and make sure there’s no hard parts on the inside.

- 35-year-old man

### Early abscess self-care treatment

Common themes of staying hydrated, eating well, using a warm compress, adequate sleep, and applying salve to the affected area were all discussed as techniques for home health care of early stage SSTIs. Notably, antibiotics and/or pursuing medical attention were not referenced during this stage of SSTI development, though they are likely clinically indicated.

Little things like drink a lot of liquids, make sure you sleep every night. Make sure you get enough sleep, drink liquids, eat regularly. These are key points.

- 35-year-old man

My thing, I swear by it, is draw-out salve.

- 37-year-old woman

It all depends. If you catch it right off the bat, you can get some type of a hot pad or something, and put it on there. You continue to do that procedure.

- 52-year-old man

### Late abscess self-care treatment

Participants described how they obtain antibiotics from non-traditional sources.

I sold antibiotics

- 37-year-old woman

You can go into the aquarium, the fish store. They have antibiotics there that are for the fish. And they will go and they will get them and they will take them.

- 37-year-old woman

Common themes of keeping the wound clean, changing dressings regularly, and packing the wound were discussed across participants.

Keeping it clean, washing the wound, and making sure its dry. Also using a certain type of packing, like a gauze with iodine, it’s used to pack the inside of a wound with antibiotic ointment. Basically, changing your bandage 1 to 2 times a day gauging on the discharge.

- 42-year-old man

I use wet-to-dry bandages with another bandage on the top of it. It’ll help it drain it and it will debride the dead tissue when you pull it out three times a day.

- 42-year-old woman

Participants identified needle aspiration as a self-care technique for SSTI.

A lot of people will take needles and put them in there and try to drain the fluid out like that.

- 35-year-old man

And sometimes they’ll poke it with a needle and get it drained.

- 42-year-old woman

Participants discussed incision and drainage techniques when treating SSTIs that reach the point of forming an abscess. This was viewed as a daunting, but sometimes necessary task.

I think it’s called expression? To open it up, to try and get all the material out. I can’t remember the term … Irrigate. People use a large syringe, preferably with the cleanest water they can get.

- 42-year-old man

I would lance it and squeeze it. I pull the skin apart as I’m pushing down on it. I push down on it really hard and pull so that the hole opens up, and then continue pushing and going like this [kneading motion] if it was on my arm, wiggling it.

- 35-year-old man

Because little blood clots still plug up the hole and by wiggling you get them to move out of the way so that the little guy will go through and open the hole up, and the bigger stuff will come out, and then everything drains out. And that’s without lancing, I meant. And then I cut it, so I can see what’s underneath, and peel the skin back, and clean it off with peroxide and then find a way to pop the core out. The core is, the core. If you don’t get rid of the core, you don’t get rid of the infection.

- 35-year-old man

… some people are pretty brave and they’ll either drain it themselves or … I mean, in all honesty, you don’t really want to go to the hospital because whether it’s a hospital or not, like they uh, they tend to uh, look differently on you when you come in with an abscess.

- 37-year-old man

### Traumatic past experiences within the formal medical system

Many participants described how past negative experiences with health care providers are a strong motivation for delaying care-seeking for their SSTIs. These negative experiences ranged from discomfort and judgment to psychological and physical trauma.

He was mean and cold and short with me. I felt like he didn’t want to touch me.

- 37-year-old woman

I didn’t get treated very well at all. It was almost like that attitude that ‘You put yourself here. What are you going to expect us to do? I hope you do suffer’

- 42-year-old woman

Incorrect treatment, when they cut a larger part than they needed to, mainly to increase pain...

- 42-year-old man

...and literally made it worse than it had to be. You know, inflicted as much pain as they could. By not administering anesthetic and not enough of what they did, and then just grinding away at you in a way that was very unprofessional. ‘Maybe this will teach you a lesson about using drugs’.

- 49-year-old man

## Discussion

PWID report a general reticence to seek formal medical care for SSTIs, despite a thorough knowledge of SSTI and associated risk of morbidity and mortality. This leaves many PWID in a position to engage in self-care strategies. Participants accurately describe the risk factors, symptoms, and natural history of SSTIs, noting progression cellulitis that may be self-limited to an abscess that requires intervention. Participants describe a range of self-care techniques. Non-invasive techniques for early cellulitis include applying hot compresses, applying salves, “whole body” health such as hydration and rest, and procuring antibiotics from non-medical sources. Once the SSTI formed into an abscess, more invasive self-care techniques were considered necessary. Invasive techniques include needle aspiration and incision and drainage procedures, performed on oneself or by another non-medical person. Many participants also expressed familiarity with effective techniques for wound care once an abscess is opened.

Participants generally had robust knowledge and experience with SSTIs. This creates an opportunity for partnership, empowerment, and knowledge exchange between PWID and the healthcare community. Harm reduction agencies and healthcare professionals should create open dialogs with PWID to learn from these communities and subsequently develop workshops and printed information to propagate this knowledge and address any misconceptions that may exist. One important conceptual deficit we identified was defining specifically when an SSTI has progressed to the point when it needs prompt medical intervention. This critical point is acknowledged by the participants, but not clearly defined. Given the common hesitancy in seeking formal care, this is crucial, and education efforts should focus on identifying serious SSTIs early.

Participants also reported a variety of techniques to treat themselves in the absence of medical care, ranging from non-invasive to invasive procedures. Generally, the non-invasive techniques, including warm compresses, applying salves, clean bandages, and optimizing general health while fighting an infection are in line with common medical recommendations, and these should be encouraged without reservation. Peer educators should be empowered to facilitate workshops, provide material resources, and educate their communities on these topics. Receiving this education from a trusted peer can propagate this knowledge, engender trust, and provide linkage to harm reduction agencies and the health system, as has been demonstrated with other disease processes in communities experiencing barriers to care [21]. The prompt identification of severe or worsening infection that requires prompt medical care should be emphasized in these teachings. These peer educator/advocates must be provided with the tools and knowledge to do so confidently and effectively. These recommendations should also be provided with the caveat that it is always best to seek medical attention if one is able to do so, and these techniques alone will not cure all SSTIs.

Due to the marginalized status of PWID and their structural barrier to care, self-directed and peer-based care has been a long-standing coping strategy for SSTIs [9, 20]. Levin noted the long history of public demand for self-care among the general population and provides a sociological and ethical framework to understand this [16]. In line with harm reduction philosophy and emphasizing patient autonomy, they underscore the importance of peoples’ independence and ability to define their own “risk mix.” They further state that “people’s integrity in making health decisions and their ability to perform on their own behalf take precedence over any and all existing professional values of risk reduction and disease cure … health professionals will have to reorient their perspectives on health, with reduced primacy” [16]. In addition, task shifting to peer health workers (PHW) is an ongoing debate in low-and-middle-income countries with insufficient medical staffing. In addition to expanding capacity, PHWs can also bridge the cultural gap between the patient and the provider. This occurs because the peer health workers are fluent in the vernacular of their patients, they are the patient’s first point of contact, easing them into the difficult to navigate norms of health care setting, and can educate the health care provider on providing culturally appropriate care to the patient [18]. Peer health workers are another resource that should be considered in addressing the disparities in care for PWID.

More invasive self-care techniques, including taking non-prescribed antibiotics and incising abscesses, were also reported with surprising frequency. This gives rise to myriad concerns, including increasing antibiotic resistance, allergic reactions, antibiotic-associated diarrhea, and surgical complications including hemorrhage and seeding deep space and orthopedic infections. However, given the ubiquity of these techniques for SSTIs, and the general mistrust in the medical system among this population, simply discouraging self-care will not likely be effective, especially in the absence of feasible alternatives. Chastising these behaviors would likely be detrimental to the already fragile relationship between healthcare providers and PWID. The optimal way to dissuade these risky behaviors is to make them unnecessary: PWID should have access to healthcare. Unfortunately, achieving this is slow and halting. Mending the relationship between PWID and healthcare providers requires significant effort. Healthcare providers should be educated on the needs and perspectives of PWID, and PWID should be provided with resources for navigating a health system that is often hostile to them. This is additionally challenging due to the disadvantageous power dynamics that PWIDs are subject to. Alternatively, creating access to health care providers in low-threshold and unconventional venues, including syringe exchanges, street outreach, free clinics, and homeless shelters/housing, may improve access and obviate the need for these risky self-care behaviors. When coupled with compassion and humility, these approaches may allow otherwise guarded patients engage in trusting and productive relationships with healthcare providers in spaces that are safe and supportive to them. This would not only benefit these individual persons, but also build trust in these communities and reduce the need for more costly and morbid intensive care downstream. However, these resources are currently scarce in most regions and are not comprehensive in availability or scope where they are present. These resources require substantial investment and should be prioritized as an important component in addressing this issue, but this is not likely a viable, universal, or timely solution in the near future.

Finally, in the absence of tangible alternatives, and in the spirit of harm reduction, PWID should be provided with resources to perform these self-directed interventions as safely and effectively as possible. The reality of these behaviors should be acknowledged and discussed openly, and not treated as taboo or blindly discouraged. There are certainly risks conferred by the self-care practices that PWID are forced to resort to. However, these risks are not taken lightly by PWID; they are weighed against the risk of inaction and worsening infections, which is well known in these communities. The resourcefulness of these communities should be recognized, and this discourse should first be viewed as an opportunity to learn from these lived experiences, and secondarily as an opportunity to provide education on best practices, and also the potential futility and harms of these self-directed approaches. In line with this aim, harm reduction agencies could provide PWID-friendly antibiograms describing the types, dosages, and durations of antibiotics that are helpful for SSTI in that region, while also urging caution, recommending physician consultation, and describing risks of using inappropriate or inadequately dosed antibiotics. Similarly, PWID should be educated on draining abscesses safely and effectively, while being encouraged to seek formal care if they are able. Harm reduction agencies could provide educational materials that include aseptic methods, incision techniques, wound exploration and packing, and hemorrhage control. They should also describe high-risk areas and presentations that should only be addressed by a medical professional (i.e., abscesses in the neck, abdomen, hands, and groin, and wounds concerning for necrotizing soft tissue infections), and provide guidance on addressing these through formal healthcare channels. They could also provide equipment including antiseptic solution, gloves, scalpels, sterile gauze, and even topical anesthetics. These resources should be provided with the caveat that medical attention is always the best course of action, while acknowledging that this is not always feasible for many PWID. Given that these practices are already common, employed in the absence of adequate training and supplies, and few feasible alternatives exist for many PWID, it is unlikely that supplying resources and education will increase the incidence of self-care or the theoretical harms associated with them. Conversely, these educational interventions and resources to support PWID will almost certainly decrease the harms associated with these self-care techniques, even if the rates remain constant. An open dialog regarding the reality of these self-care techniques may be a potent tool in decreasing the risk of complications due to improper care, and ultimately may decrease the prevalence of invasive self-care by creating new relationships and avenues to achieve formal care. Empowerment and education is the responsible approach to minimizing harm and improving outcomes for this vulnerable population that is too often excluded from formal medical care.

Understanding and addressing the root of why PWID partake in self-care is essential. One key factor to delay is past or perceived negative experiences arising from contact with medical providers. Discrimination, unaddressed pain, fear of withdrawal, and stigma are all reasons why PWID delay seeking care from medical professionals [1, 26]. The most important strategy health providers at all levels can employ is to reach out to spaces that are low-threshold for PWID or work to educate other healthcare providers and change their local culture to create inclusive environments and provide positive medical experiences for PWID. Addressing pain concerns and staving off opioid withdrawal in a clinical setting, while treating this population with compassion and dignity, is paramount to improving trust and subsequently access for this marginalized population.

This study does have some limitations and is primarily exploratory and hypothesis generating. First, this is a secondary analysis of qualitative data obtained for a separate, but related study. Ideally, the authors would have conducted subsequent interviews, further exploring the specific topic of interest and reaching saturation in these communities. Second, the sample was largely white and male. Future investigations should purposively seek the perspectives of people of all genders, races, and ethnicities. The study was also limited to two metropolitan areas, and while they are distinct from one another, they do not represent all communities of PWID. Particularly in nuanced topics like this, more geographic diversity would benefit the generalizability of the findings. Third, this study is a secondary analysis of qualitative themes that emerged during the initial data collection. However, given that the initial focus of this project was not self-care per se, saturation was not reached on this theme and many novel codes continued to emerge in retrospective analysis. SSTI self-care is individualized and multifaceted and achieving saturation would likely require a much larger cohort, specifically targeted for diversity of self-care experiences. Unfortunately, the researchers did not have the ability to expand the qualitative cohort at this stage of the project, so only our initial exploratory findings are presented here. Lastly, this study relies heavily on participants’ recollection, and is susceptible to recall bias, particularly for these traumatic and emotionally charged events.

## Conclusion

In our sample of PWID, there is accurate working knowledge of the stages of SSTI pathophysiology and treatment options. Despite appreciating the potential severity of this illness, the reticence to pursue formal care due to previous firsthand or secondhand negative health care experiences is ubiquitous and concerning. Therefore, many PWID take SSTI treatment into their own hands. This knowledge and many of these behaviors appear empowering to participants, and may be beneficial. However, others pose substantial risk, such as unsafe surgical procedures and inappropriate antibiotics usage. The need for self-care could be decreased by providing low threshold care and building trusting relationships between PWID and healthcare providers, but this is not expeditious or universally available. Acknowledging the reality of these behaviors and embracing a harm reduction approach, educational interventions should be tailored towards spreading and improving upon this working knowledge through peer teaching and materially supporting PWID. Harm reduction agencies and facilities that create safe spaces for PWID are vital in empowering this marginalized population and providing education about SSTI and tools for self-care. Further research should focus on the effectiveness of low threshold SSTI care, educational outcomes and behavior changes in PWID educated on SSTI self-care, and clinical outcomes for those engaging in self-care for SSTI.

## Abbreviations

CA: California
HBM: Health belief model
IDU: Injection drug use
IRB: Institutional review board
MA: Massachusetts
PWID: People who inject drugs
PWIH: People who inject heroin
SSTI: Skin and soft tissue infections
US: United States
USA: United States of America
